# Supplementary material for: The discovery of novel noncoding RNAs in 50 bacterial genomes
Source: Nucleic Acids Res. 2024 Apr 22;52(9):5152–65. doi: 10.1093/nar/gkae248 (PMC11109978; doi:10.1093/nar/gkae248)

sRNAC-34-1-NC_002936_3_IGR_12_1394982_1395431.sto.r2r

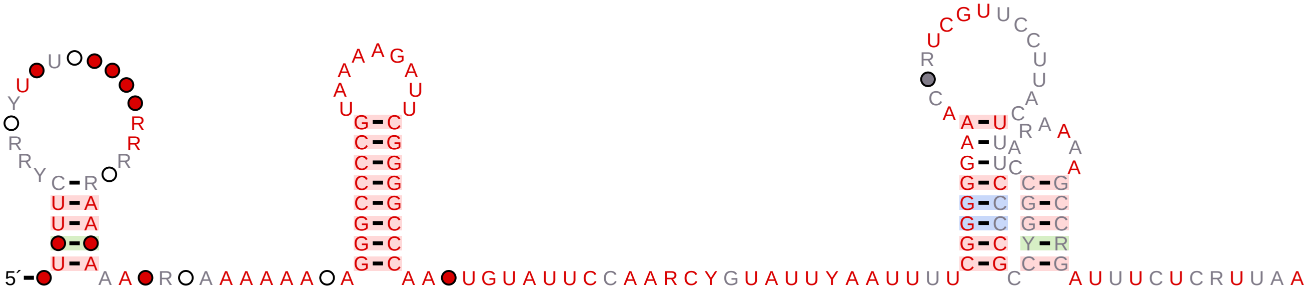

Supplement: gkae248_Supplemental_Files [file gkae248_supplemental_files.zip › Narunsky 50 Genomes RNA Motifs Files.zip!/Publication/34-D_mccartyi/Figures/sRNAC-34-1-NC_002936_3_IGR_12_1394982_1395431.sto.pdf]
